# Preparation of Modified Montmorillonite—Plant Fiber Composite Foam Materials

**DOI:** 10.3390/ma12030420

**Published:** 2019-01-30

**Authors:** Qifeng Chen, Xinya Du, Guangxue Chen

**Affiliations:** State Key Laboratory of Pulp and Paper Engineering, South China University of Technology, Guangzhou 510640, China; qfchen@scut.edu.cn (Q.C.); 15603064069@163.com (X.D.)

**Keywords:** plant fiber composite foaming, ODBA, OMMT, sound absorption

## Abstract

In the present study, plant fiber foam materials have significant differences in density compared to conventional plastic foam materials. In view of the current problems of fiber foam materials, the montmorillonite (MMT) organically modified by octadecyl-dimethyl-benzyl-ammonium chloride (ODBA) was added to the preparation of composite foam materials by optimizing the existing formula. The properties of organic montmorillonite (OMMT) and the prepared composite foam materials were characterized by scanning electron microscopy (SEM), X-ray diffractometry (XRD), and a standing wave tube sound absorption tester. The results showed that the pore size inside the plant fiber foam materials with the addition of OMMT was more uniform and arranged more closely and orderly. In addition, when the OMMT was added to 0.1 g, the density of the prepared OMMT-bagasse composite foam materials reached its lowest point of 0.079 g/cm^3^, which was shared with high foam materials.

## 1. Introduction

In recent years, with the explosive development of e-commerce platforms and take-out industries, the demand for plastic foam materials has also exploded. Although the government has issued a series of restrictive policies on ‘white plastic garbage’, including ‘plastic limit order’, the 2017 China Express Industry Green Packaging Development Status and Trend Report [1] pointed out that usage of plastic bags has grown from 3 billion to 22 billion since the ‘plastic limit order’ implementation. Therefore, Plastics Processing Industry Technology Progress 13th Five-Year Development Guidance Opinions [2] proposed to increase the research and development of energy-saving and environmentally friendly products, accelerate the pace of catching up with foreign advanced technology and production equipment, and further promote the plastics manufacturing industry reform and transformation. A series of actions and policies have indicated that searching for a green material to replace plastic foam products has become a priority.

Foreign researchers use polyvinyl alcohol (PVA), polylactic acid (PLA), and other mainstream adhesion aids as basic additives to explore their composite properties with natural high molecular polymers, such as various plant fibers and starch, and other artificial polymers. The composite performance of materials is much better than that of domestic-related research. Sony Company’s Tsutomu Nojuchi [3] used waste paper fiber as the main raw material, mixing a certain proportion of micro-polyethylene plastic pellets into modified starch, foamed by water vapor, to prepare foam materials where the compression stress was 0.5 times that of ordinary polypropylene foam. Ganjyal [4] acetylated ordinary corn starch, adding the obtained cellulose after delignifying by strong alkali, and used ethanol reagent as a foaming agent and talc as a nucleating agent. Meanwhile, material properties were explored by using different concentrations of cellulose. As a result, the compatibility between the plant cellulose and the corn starch is excellent, and the low concentration of cellulose can effectively enhance the physical properties of the foam materials. However, when the concentration reaches 10%, it has a negative impact on the density of the material and the expansion ratio.

Although domestic research started late, many research achievements and breakthroughs in key technologies have been achieved. Zhang of Jiangnan University [5] used waste corrugated board as the main raw material, modified the fiber with NaOH, and used polyvinyl alcohol (PVA) as the main adhesive to prepare foam materials by low-power microwave foaming technology. The results show that the initial water content of the pulp material is between 90% and 95%, the foam materials have the lowest density and the highest expansion ratio, and the optimum ratio of each ingredient is determined by experiments. Lu et al. [6] used waste paper fiber and corn starch as the main raw materials and Na_2_CO_3_-citric acid as the foaming agent, and explored physical properties of the foam materials. Finally, the foam materials with a porosity of 87%, a density of 0.2 g/cm^3^, and a Young’s modulus of 700 MPa were obtained. Yang [7] adopted the melt intercalation method to prepare starch/bean residue composite foaming cushioning material with different contents of MMT. The results indicate that the mechanical strength, water resistance, and compression strength of the composite improve significantly when the mass fraction of montmorillonite (MMT) is 5%.

MMT is a type of clay; it has a unique and natural nanostructure, with a slice scale of nanoscale. Therefore, it is often used in the preparation of nanocomposites. MMT is similar to the effect of nucleating agents, which can increase the number of cells formed in the unit volume during the foaming process. In addition, the layered structure of MMT promotes it to uniformly adsorb on the skeleton structure of the fiber web, and then it becomes the skeleton support structure of the fiber web, so that the cells of the obtained foam materials increase and the pore diameter becomes smaller [8].

In this work, the formulation and reaction conditions were further optimized based on previous studies [5]. Furthermore, the MMT was modified and added to the foaming process, where bagasse fiber and corn starch were used as the basic raw materials. Finally, the composite foam materials were prepared by microwave foaming, and the influence of the foaming process condition and the content of various reagents on the properties of the foam materials were investigated. The purpose is to provide a theoretical basis for realizing the industrial production of green degradable materials instead of plastic foam materials as soon as possible.

## 2. Materials and Methods

### 2.1. Materials

The bagasse was purchased from local farmers (Yizhou, Guangxi, China), corn starch was bought from Tianjin Kemiou Chemical Reagent Co., Ltd. (Tianjin, China), and sodium tetraborate and calcium carbonate (CaCO_3_) were purchased from Guangzhou Fuchen Chemical Reagent Co., Ltd. (Guangzhou, China); the above reagents were of analytical purity. MMT K-10 was purchased from Maclean (China) Chemical Reagent Co., Ltd. (Guangzhou, China). MMT is a type of natural mineral of layered silicate with a specific surface area of 240 m^2^/g and a molecular weight of 30.06900.

Polyvinyl Alcohol (PVA) 350 was purchased from (Wokai) Sinopharm Group (Shanghai, China); ammonium bicarbonate was purchased from Guangzhou Fuchen Chemical Reagent Co., Ltd. (Guangzhou, China); glycerol was purchased from Shanghai Chemical Reagent Co., Ltd. (Shanghai, China); and octadecyldimethylbenzylammonium chloride (ODBA) was purchased from Aladdin Chemical Reagent Co., Ltd. (Shanghai, China); the above reagents were of national standard purity.

### 2.2. Preparation of Corn Starch-Bagasse Foam Materials

**Figure 1:**
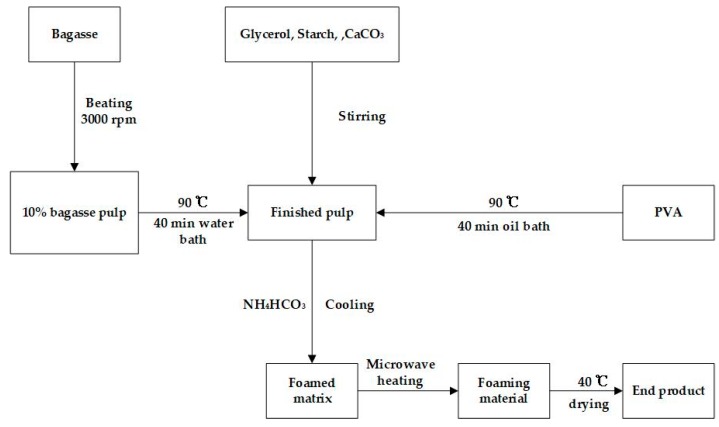
Foaming process diagram.

Some bagasse pulp board was immersed in water for about 2 h and then beat for 3000 rpm after spalling; then, some bagasse was placed into a sealed bag at 90 °C, placed in a water bath for 40 min, and kneaded every 10 min. A certain amount of PVA was put into a round bottom beaker and heated in a 90 °C thermostatic heating magnetic stirrer (DF-101S, Gongyi Yuhua Instrument Co., Ltd., Zhengzhou, China) for 40 min until completely dissolved. A small amount of starch was mixed with water with a ratio of 1:3 and stirred constantly in a water bath at 70 °C until it started gelatinizing. The bagasse pulp was mixed with PVA solution and stirred uniformly, then glycerin, calcium carbonate, borax, and starch solution were added. A certain amount of NH_4_HCO_3_ was added after mixing well. When all were mixed uniformly, the obtained mixture was poured into the mold and heated for a certain time in a microwave oven (M1-L213B, Guangdong Midea Electric Co., Ltd., Guangzhou, China) using 700 W power. Finally, the foam materials were obtained after drying for 12 h when the temperature of the drying oven was set to 40 °C. The specific foaming process is shown in Figure 1.

In this experiment, NH_4_HCO_3_ was used as a foaming agent. NH_4_HCO_3_ was easily decomposed by heating, and the decomposition equation was as follows:

(1) $${\mathrm{NH}}_{4}{\mathrm{HCO}}_{3}\overset{\mathrm{Heating}}{\to }{\mathrm{NH}}_{3}↑+{\mathrm{CO}}_{2}↑+H_{2}O↑$$

Water was used as a medium for microwave heating. Most of the water in the raw material will be evaporated. As the system was no longer closed during the process of changing from liquid phase to solid phase, NH_3_ and CO_2_ will eventually escape from the material. This was the foaming mechanism of this experiment.

### 2.3. Preparation of OMMT

A certain amount of MMT K10 was weighed into a beaker, adding a quantitative amount of 2 mol/L hydrochloric acid solution and adjusting the PH of the solution to 3. ODBA was added into the stirring turbid solution. After heating in an oil bath at 80 °C for 40 min, it was then put in a vacuum oven for one night. Finally, the dry OMMT powder was obtained by grinding fully.

**Figure 2:**
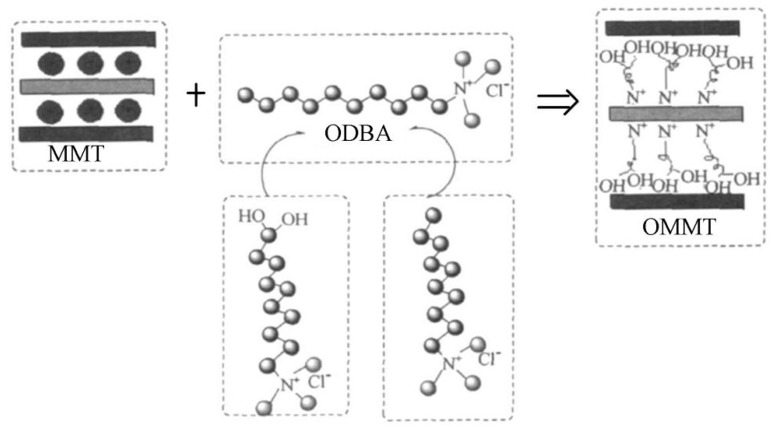
Modified schematic of montmorillonite (MMT).

The intercalation method was used in this experiment. It was an important method for preparing polymer/inorganic nanocomposites. Many inorganic compounds, such as silicate clay, phosphates, graphite, metal oxides, disulfides, and phosphorus trisulfide complexes, have a typical layered structure and can be embedded in organic matter. The intercalation method is simple in terms of process and rich and inexpensive in terms of raw materials. The usage of the intercalation polymerization method can improve the mechanical properties of materials reduce costs, and the laminar structure and interface development of MMT can increase the porosity and hygroscopicity of fibers [9,10]. In this experiment, an organic quaternary ammonium intercalation agent [11] was used to modify natural MMT, in order to improve the adsorption of MMT on cellulose and chemical additives. The reaction mechanism is shown in Figure 2. The methods for MMT modification included cation exchange reactions, silane grafting, and polar polymer adsorption [12]. In this work, the cationic long carbon chain of the organic quaternary ammonium salt was inserted and covered between the layers of the natural MMT by the ion exchange reaction.

## 3. Result and Discussion

### 3.1. The Effect of Reaction Conditions on the Properties of Foam Materials

#### 3.1.1. The Effect of Initial Moisture Content of Materials on Foam Materials

The initial moisture content of the material, which was the mass of water in the raw material, as a percentage of the total mass of the raw material, was as follows:

ω% = m_water_/m_total_ × 100% (2)

The foaming ratio depended not only on the decomposition of the foaming agent but also on the evaporation of water. The initial moisture content of the material directly affects the foaming ratio. The material with high initial moisture content had a higher volume before foaming, which resulted in a decrease in the volume ratio, before and after foaming. In addition, the rapid gasification of water molecules broke through the bubbles, causing the bubbles to rupture and the foam materials to collapse. Therefore, the different initial moisture contents were obtained by adjusting the proportion of water in the total material. The density of the foam materials with different initial moisture contents was calculated by weighing the mass of the foamed material and measuring the volume multiple times by the vernier caliper. In the end, the effect of initial moisture content on the foaming density of the material was explored. The results are as follows.

**Figure 3:**
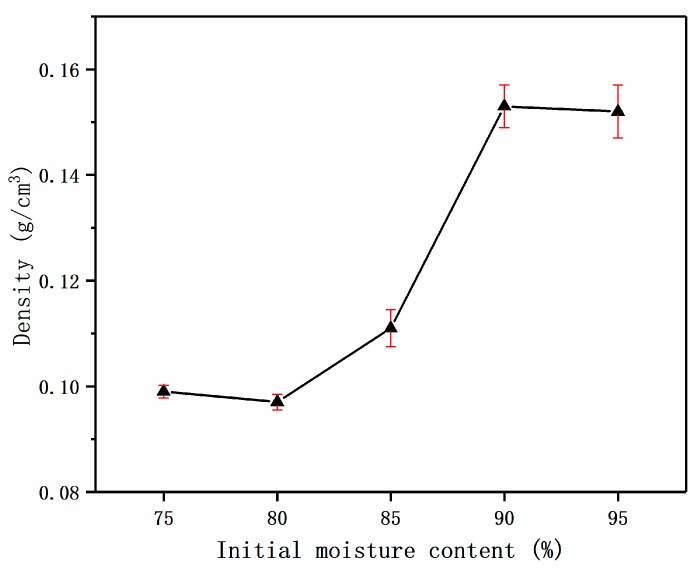
Effect of different initial moisture content on density.

According to Figure 3, when the initial moisture content of the material was 80%, the density reached a minimum of 0.097 g/cm^3^. When the initial moisture content of the material was 75–80%, the density of the material showed a weak downward trend. This was because the concentration of adhesive PVA in the bubble body was excessive. The external resistance was slightly larger than the force of generating the internal bubble core, resulting in some late bubble core collapsing in the growth stage. When the initial moisture content of the material reached 80%, the internal and external force were balanced in the stable period, and the retention of the bubbles was ensured to the utmost extent. When the initial moisture content of the material exceeded 80%, the thermal motion of water molecules was more severe by the microwave, due to the increasing of the water content in the material. That meant the force of the pore wall greatly increased, and a large number of pores ruptured in the middle of the reaction. Some of them were broken down into smaller vesicles remaining in the material, and some escaped from the material, resulting in the density of the final foam materials increasing.

#### 3.1.2. Effect of PVA Content on Properties of Foam Materials

According to the adsorption theory [13], the mechanism of action of PVA as an adhesive was mainly the intermolecular force of the adhesive system and the force of hydrogen bonding. When the distance between the PVA molecule and the bagasse fiber was less than 10 nm, the adsorption force was generated, and the position of the PVA molecule and the fiber surface reached relatively stable state. Therefore, the amount of PVA determined the density of the foam materials to some extent. The foam materials were prepared by changing the amount of PVA and measuring the density of the obtained foam materials. The relationship between the two is as follows.

**Figure 4:**
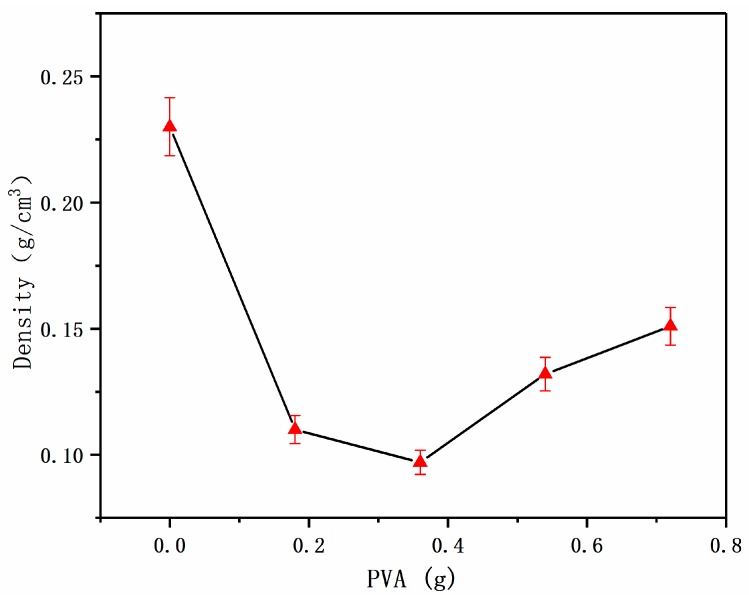
Effect of polyvinyl alcohol (PVA) dosage on density.

It can be seen from Figure 4 that the density of the foam materials was smallest when the amount of PVA was 0.36 g. When PVA was not added, the density of the material was highest. That was because the viscosity of the internal fluid was low, and the bubbles persisted to expand at the beginning of the growth period. Finally, several bubbles were aggregated into large bubbles, and the large bubbles were easily broken in the late growth stage with temperature increasing. Moreover, the surface of the bubble body cannot form a film without the adhesive, which caused the gas to escape from the bubble body and led to the increase of density. With the increasing amount of PVA, the viscosity of the bubble body also increased, and for the bubbles, the external resistance became excessive, causing the pores to collapse, resulting in gradual increase in density.

#### 3.1.3. Effect of Reaction Time on Properties of Foam Materials

**Figure 5:**
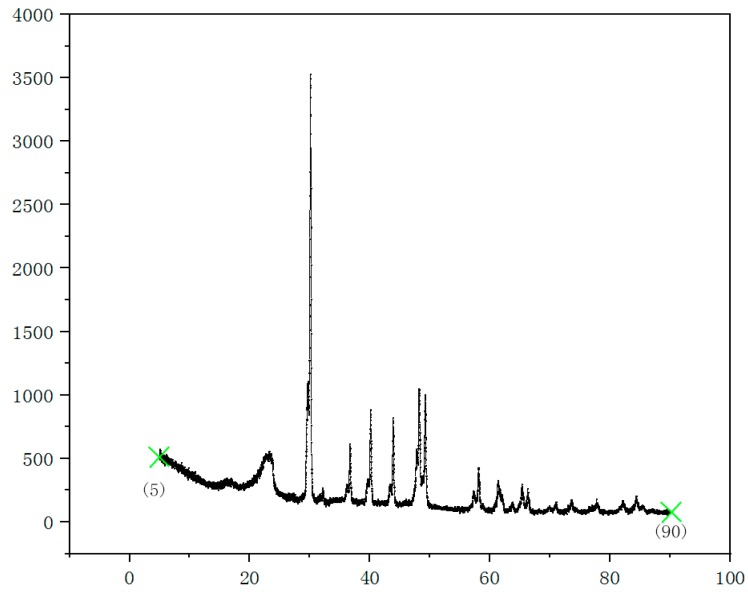
X-ray diffractometry (XRD) pattern of foam materials.

Because of the microwave process, intense carbon–oxygen organic compounds, such as fibers, were easy to carbonize and even burning. Therefore, precise control of the reaction time was essential for the preparation of the foam materials. Since the effect of foaming time on density was not intuitive, the residual stress of the foam materials was chosen as a measurement index. The residual stress of the foam materials was investigated by a D8 Advance X-ray diffractometry (XRD, Bruker, Billerica, Germany). In general, the residual stress referred to existing stress at a macroscopic level. The macroscopic residual stress expressed in the X-ray diffraction spectrum was the peak shift. The residual stress can be obtained by measuring the displacement of the diffraction peak of the sample [14]. Experimentally discovered, the foaming body with 80% moisture content forming needed at least 16 minutes in the microwave environment of 700 W, and carbonization occurred in the center of the bubble after 23 min. Ensuring the material forming and without carbonization, the experiment was set up for 18 min, 19 min, 20 min, 21 min, and 22 min. A force of 10 N was applied to the surface. The results are shown in Figure 5 below.

The relevant data was calculated using Jada software 6.5 (Materials Data Inc., Aubrey, TX, USA). Jade 6.5 offered a variety of methods for determining peak positions and was equipped with stress calculations. The results are counted in Table 1 below.

As shown in Figure 5, the surface residual stress of the foam materials was smallest at the reaction time of 20 min. Within 18 to 19 min, the tendency of the peak width to become smaller was obvious. When the reaction time exceeded 20 min, the change of peak width was slight. That was because the water inside the material had been completely evaporated. The pores had experienced a complete stabilization period, so the residual strain was low.

### 3.2. Characterization of OMMT

#### 3.2.1. SEM Analysis

**Figure 6:**
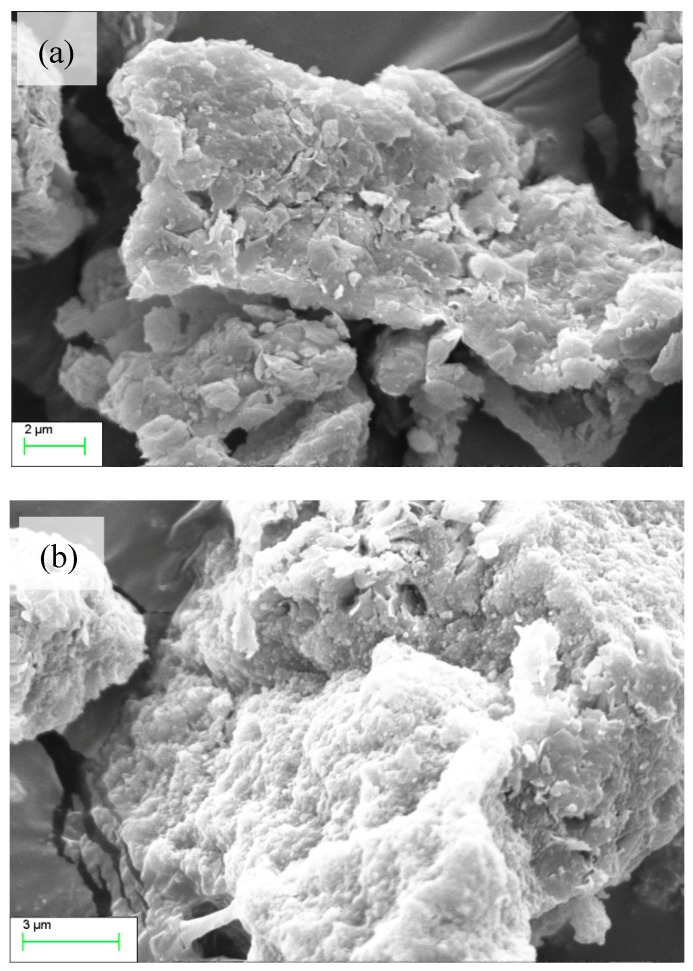
The scanning electron microscopy (SEM) of MMT (**a**) and organic montmorillonite (OMMT) (**b**).

Figure 6a shows natural MMT crystals were generally an irregularly sheet structure, belonging to monoclinic crystals, and the surface structure was relatively flat, showing orderly micron-level clumps and no obvious curl. Figure 6b is the scanning electron microscope (Hitachi S-3700N, Suzhou Sainz Instrument Co., Ltd., Suzhou, China) image of OMMT. The surface of the OMMT is still flat, while the clumps have been obviously dispersed. This indicates the charge affinity between the layers was reduced and the ordered arrangement was destroyed. In other words, the OMMT interlayer spacing was enlarged.

#### 3.2.2. XRD Analysis

**Figure 7:**
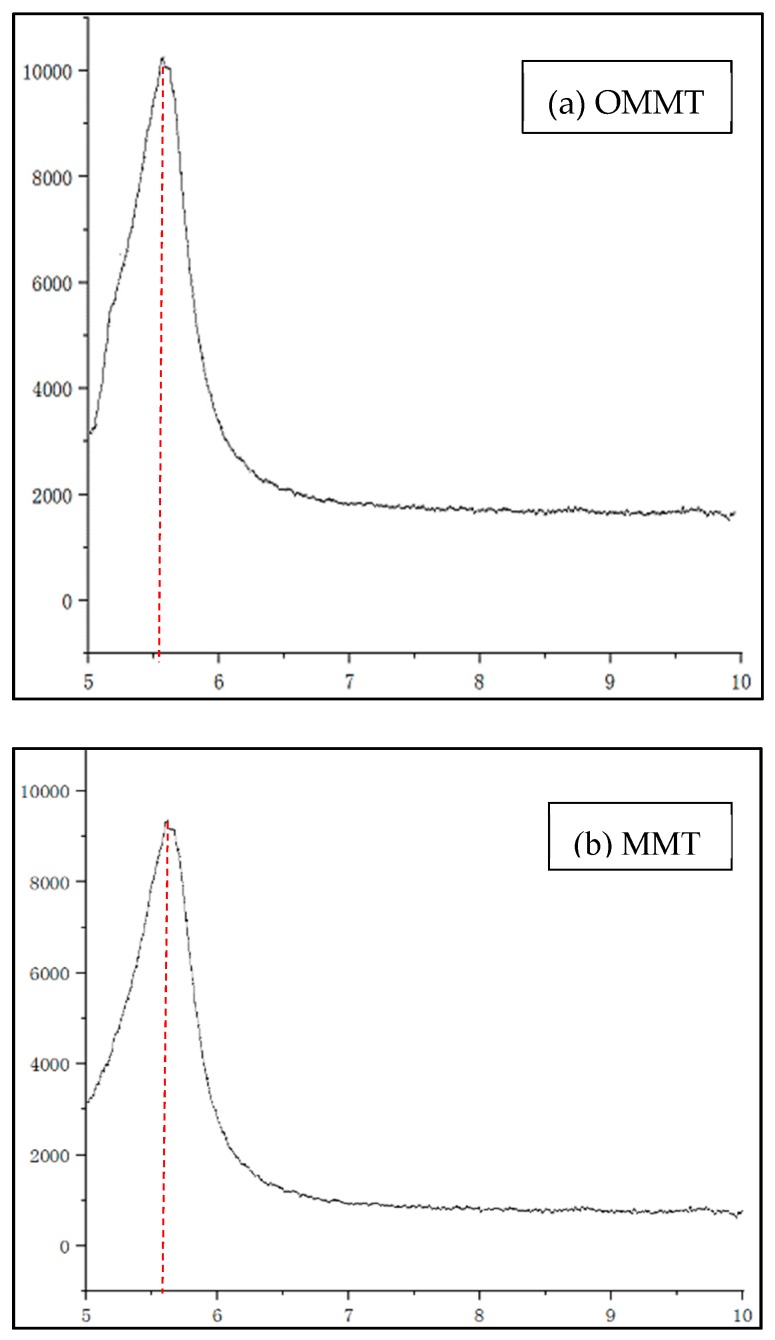
XRD patterns of OMMT (**a**) and MMT (**b**).

Figure 7 shows the comparison of XRD images of OMMT Figure 7a and natural MMT Figure 7b. The organic MMT intercalation was tested by the wide-angle X-ray diffractometer. The testing conditions of wide-angle X-ray diffractometer were Cu electrode, radiation voltage of 40 KV, radiation current of 40 mA, and scanning range of 1°–15°. When the intercalation agent inserted the MMT layer, causing the interlayer spacing increase, the X-ray diffraction characteristic peak of the MMT shifted to a low angle [11]. From the curve, it can be seen that the angle of Figure 7a became smaller than in Figure 7b. According to the Bragg formula, the interlayer spacing of MMT can be calculated to be 1.2637 nm, and the layer spacing of OMMT was 1.5462 nm.

### 3.3. The Effect of OMMT Dosage on Foam Materials

#### 3.3.1. The Effect of OMMT Dosage on Surface Structure of Foam Materials

**Figure 8:**
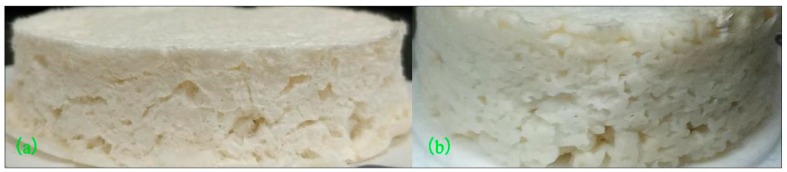
Comparison image of the basic (**a**) and OMMT (**b**) foam materials.

Figure 8 shows the picture of the foam materials before and after adding OMMT. In order to investigate the effect of OMMT dosage on the properties of foam materials, this experiment was based on the optimized formula and experimental conditions. The optimized formula ratio was absolute dry bagasse pulp:polyvinyl alcohol (PVA):glycerol:NH_4_HCO_3_:corn starch:CaCO_3_:sodium tetraborate = 9.6:0.36:1.14:0.3:0.3:1:0.1, and the optimized reaction conditions were initial moisture content of 80% and microwave power of 700 W for running 20 min. The density of the foam materials was used as the object of analysis. The experimental results are as follows.

As can be seen from Figure 8, the (b) foam materials of adding the OMMT had more and smaller pores than (a) without the addition OMMT. Under the same conditions, (b) was thicker than (a). That was to say, the foaming ratio of (b) was higher than that of (a). This may be due to the layered structure of OMMT uniformly adsorbed on the skeleton structure of the fiber web. The OMMT acts similarly to the effect of nucleating agents, which can increase the number of pores formed in the unit volume during the foaming process.

#### 3.3.2. The Effect of OMMT Dosage on the Density of Foam Materials

**Figure 9:**
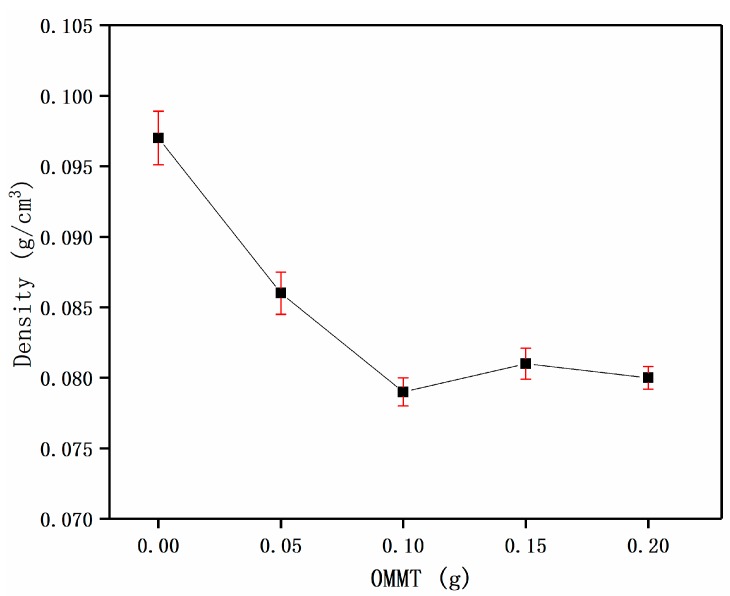
Effect of the dosage of OMMT on foam materials.

According to Figure 9, when the OMMT dosage was increased, the density gradually decreased. When the amount of addition was 0.1 g, the density of the composite foam materials reached a minimum of 0.079 g/cm^3^. When the dosage of OMMT was more than 0.1 g, the material density changed very little. This may be due to the content of OMMT being too high, which causes the filler to agglomerate, affecting the internal structure and properties of the foam materials.

In summary, after optimizing the original foam formula and reaction conditions, the foam materials with the density of 0.079 g/cm^3^ were obtained, which were smaller in density and lighter in weight than the original foam materials of 0.089 g/cm^3^ [5].

#### 3.3.3. SEM Analysis

**Figure 10:**
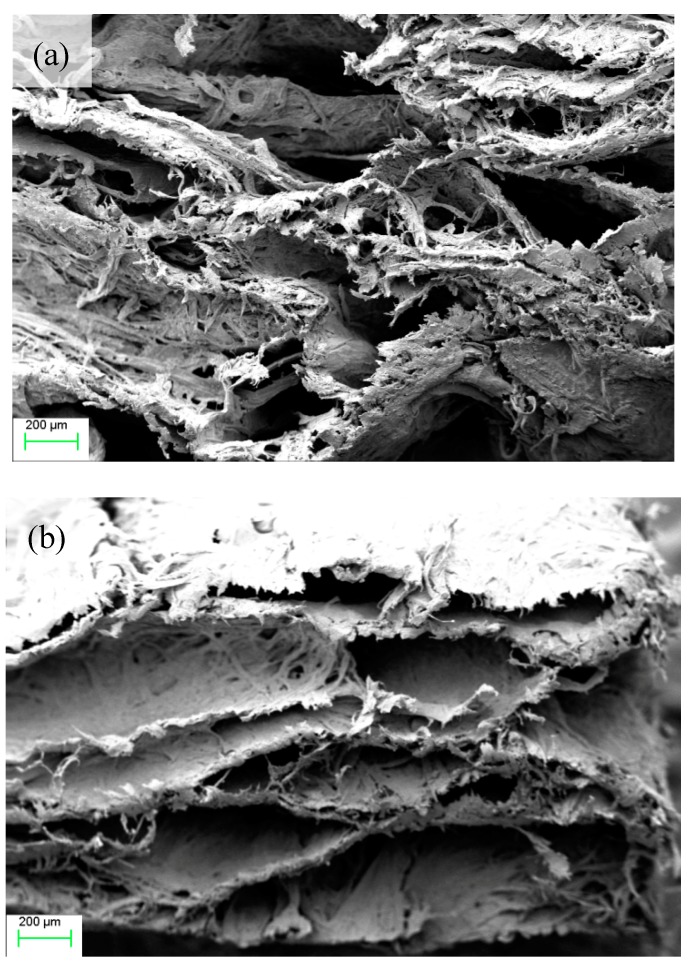
The SEM image of MMT (**a**) and OMMT (**b**).

We can see from Figure 10 that the layer distribution of Figure 10a was more disordered than that of Figure 10b. The likely reason for this was that the orderly layer structure of OMMT, combined with the fiber framework, promoted the orderly layer distribution of the whole material. The addition of OMMT improved the internal pores structure of the materials to a certain extent. The size of the pores was more uniform and compact than before, and the structure was more orderly.

### 3.4. Acoustic Performance Characterization of Composite Foam Materials

Generally, porous materials whose sound absorption coefficient exceeded 0.2 were called sound absorption materials. Their sound absorption principle was that when the sound source was close to the sound-absorbing material, the vibration wave source generated by the sound source would propagate along with the medium, making the adjacent media points vibrate. The sound absorption materials had a lot of small cells, which were arranged in a complicated manner. The complicated holes or gaps vibrated with a vibration source, causing the energy loss of the sound source, and the energy were transmitted and transformed to achieve the purpose of sound absorption [15].

**Figure 11:**
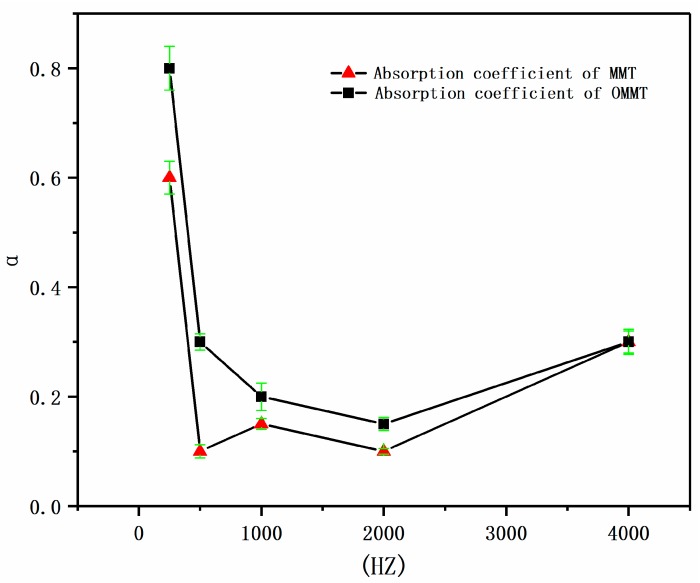
Comparison diagram of sound absorption coefficient of MMT and OMMT.

In order to further explore the sound absorption performance of the composite foam materials, the sound absorption coefficient of the foam materials before and after the addition of MMT were tested and compared. The composite foam materials were cut into 10 × 10 × 10 mm sheets and placed on a standing wave tube sound absorption tester (AWA6128A, Hangzhou Aihua Instrument Co., Ltd., China) to measure the sound absorption coefficient at different frequencies. Average sound absorption coefficient was the value obtained by averaging the sound absorption coefficient of 100–5000 Hz, which represented the sound absorption performance of the material as a whole. However, we generally used the noise reduction coefficient NRC to roughly evaluate the sound absorption performance in the language frequency range. This value was the arithmetic mean of the sound absorption coefficients of the materials at four frequencies of 250, 500, 1K, and 2K. The measurement results are shown in Figure 11.

Figure 11 shows the sound absorption coefficient at different frequencies. In the low frequency region, the sound absorption coefficient was the best, while the medium frequency and high frequency regions had slightly poor sound absorption effect. The reason for this may be that the material is too thick or the density too high. As the thickness and density of the material increased, the optimal sound absorption frequency moved toward the low frequency. It can be seen from Figure 11 that the sound absorption coefficient of the composite foam materials with OMMT was significantly improved, which proved that the OMMT had an improved effect on the sound absorbing performance of the bagasse-starch foam materials.

## 4. Conclusions

In this paper, a method for preparing the low density of the plant fiber foam materials was provided, and the existing formulation and reaction conditions were further optimized.

When the formula ratio was absolute dry bagasse pulp:polyvinyl alcohol (PVA):glycerol:NH_4_HCO_3_:corn starch:CaCO_3_:sodium tetraborate = 9.6:0.36:1.14:0.3:0.3:1:0.1, and the reaction conditions had an initial moisture content of 80% and microwave power of 700 W for running 20 min, the high foam materials with a density of 0.079 g/cm^3^ were obtained.

MMT was modified by ODBA, and the interlayer spacing of the OMMT was increased from 1.2637 nm to 1.5462 nm. The foam materials obtained by adding the OMMT had a more orderly surface structure and a more uniform internal pore size. When the amount of OMMT was 0.1 g, the dosage was the best and the material density reached the minimum. 

In addition, the sound absorption performance of the foam materials was discussed in this work. Under the same curve trend, the foam materials with OMMT had better sound absorption performance than those with MMT, which indicated that the OMMT had an improved effect on the internal pore structure of the foam materials.

## Figures and Tables

**Table 1:** Surface residual stress of foam materials at different reaction times.

Time (min)	18	19	20	21	22
Half width (mm)	21.37	19.52	8.65	10.32	12.77
Surface residual stress P	1520	1360	560	740	820
